# Extramammary Paget’s Disease of the Scalp with an Underlying Atypical Meningioma—A Case Report and Review of the Literature

**DOI:** 10.3390/life15071064

**Published:** 2025-07-03

**Authors:** Carolina Solomon, Adina Patricia Apostu, Ioana Irina Trufin, Salomea Ruth Halmagyi, Liliana Rogojan, Simona Corina Șenilă, Loredana Ungureanu

**Affiliations:** 1 Department of Radiology, “Iuliu Hațieganu” University of Medicine and Pharmacy, 400012 Cluj-Napoca, Romania; carolinasolomon12@gmail.com; 2 Department of Radiology, Emergency County Hospital, 400006 Cluj-Napoca, Romania; 3 Department of Dermatology, “Iuliu Hațieganu” University of Medicine and Pharmacy, 400012 Cluj-Napoca, Romanialoredanaungureanu08@gmail.com (L.U.); 4 Clinical Hospital of Infectious Diseases, 400000 Cluj-Napoca, Romania; 5 Department of Pathology, Emergency County Hospital, 400006 Cluj-Napoca, Romania; 6 Department of Dermatology, Emergency County Hospital, 400006 Cluj-Napoca, Romania

**Keywords:** extramammary paget’s disease (EMPD), ectopic EMPD, intraosseous meningioma

## Abstract

Extramammary Paget’s disease (EMPD) is a rare adenocarcinoma originating from apocrine glands, primarily affecting the anogenital and axillary regions. Ectopic EMPD, occurring in non-apocrine sites such as the scalp, is exceptionally uncommon. We report a case of ectopic EMPD of the scalp distinguished by its association with an intraosseous atypical meningioma, a previously unreported finding. A 70-year-old female presented with a persistent 15 cm erythematous, alopecic scalp lesion that had been misdiagnosed for a decade. Histopathological analysis confirmed ectopic EMPD, while imaging identified an adjacent intraosseous meningioma. Surgical resection was performed for the meningioma, and radiotherapy was selected as the treatment modality for EMPD. Due to its clinical resemblance to inflammatory dermatoses, ectopic EMPD is often underrecognized, underscoring the critical role of histopathology and immunohistochemical markers in diagnosis. Given its potential association with malignancies, comprehensive systemic evaluation is warranted. The high recurrence rate following surgical excision necessitates consideration of alternative therapeutic approaches, including radiotherapy, Mohs micrographic surgery, or photodynamic therapy. This case highlights the necessity for heightened clinical awareness of atypical scalp lesions and underscores the importance of thorough diagnostic assessment. Further research is needed to clarify the relationship between EMPD and other neoplasms and to optimize management strategies.

## 1. Introduction

Mammary Paget’s disease is most commonly a manifestation of the extension of an underlying breast adenocarcinoma and is typically localized to the nipple or areola [1]. Extramammary Paget’s disease (EMPD), first described by Crocker in 1889 in a case involving the scrotum and penis, is a rare, slow-growing adenocarcinoma originating from the apocrine glands in the anogenital and axillary regions [2]. Less frequently, EMPD can develop in areas where apocrine glands have low density, such as the eyelid, umbilicus, and scalp, and is referred to as ectopic EMPD [3,4,5]. Clinically, EMPD presents as a scaly, mildly pruritic, slowly enlarging erythematous lesion, often mimicking inflammatory conditions, tumors, or infections [6]. We report a case of a 70-year-old female patient with ectopic EMPD of the scalp, characterized by an unusual clinical presentation and associated with an underlying intraosseous meningioma.

## 2. Case Report

A 70-year-old Caucasian female presented with a slow-growing, mildly pruritic, erythematous, alopecic patch on the left fronto-parieto-temporal region, which had been present for approximately 10 years. Her medical family history was unremarkable. Her past medical history included hypertension, chronic ischemic heart disease, asthma, multinodular thyroid, grade I obesity, and a hysterectomy with adnexectomy for uterine fibroids. In 2004, she was diagnosed with multiple meningiomas and she underwent excision of a right frontal meningioma.

In 2021, a PET-CT scan revealed a lytic, permeative lesion in the left skull, prompting a recommendation to rule out multiple myeloma. The PET-CT examination was carried out on an Optima PET/CT 560 machine, GE Healthcare, Waukesha, Wisconsin; exposure dose 326.34 mGy × cm. A subsequent hematological evaluation excluded the diagnosis of multiple myeloma.

**Figure 1:**
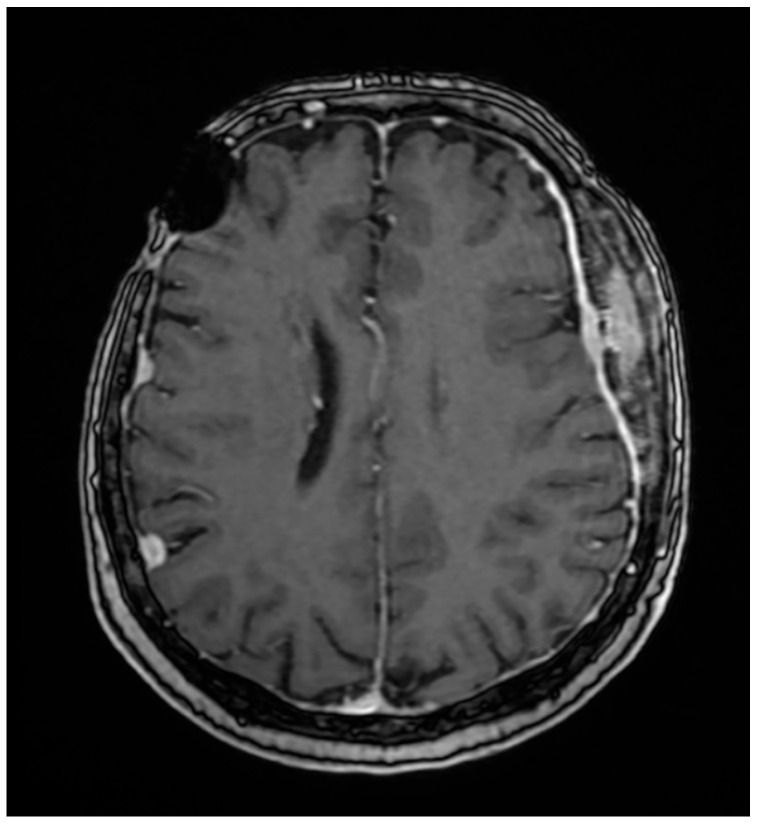
MRI examination with iv contrast media, axial 3D T1 FSPGR (14 February 2023). MRI (2023) showing a porencephalic cavity in the left fronto-temporo-parietal region, post-neurosurgical intervention. Tumor-related changes include a large central osteolytic lesion (49 × 22 × 76 mm) with irregular bone structure and soft tissue extension. Surrounding inhomogeneous osteosclerosis, diffuse meningeal thickening, and mild temporal muscle edema are noted. No contrast enhancement is seen in the porencephalic cavity. Stable multiple meningiomas are also present.

Her 2023 MRI revealed a porencephalic cavity following neurosurgical intervention, which communicated with adjacent cerebral sulci, resulting in secondary widening and perilesional gliotic changes. No contrast enhancement was observed. At the left fronto-temporo-parietal bone, tumor-related changes were noted, characterized by contrast enhancement, bone structural inhomogeneity, and extension both intracranially (extraneuraxial involvement) and into the soft tissues of the scalp. The central osteolytic area measured 49 × 22 × 76 mm (AP × LL × CC) and was surrounded by inhomogeneous osteosclerosis of the adjacent skull. The meninges appeared diffusely thickened with mild irregularities and contrast enhancement. Additionally, discrete edema was observed in the temporal muscle and adjacent fat tissue. The MRI also demonstrated stable findings of multiple right-sided meningiomas (frontal, temporal, and parietal), a left temporal meningioma, and a fronto-temporo-parietal intraosseous meningioma (Figure 1). The magnetic resonance examination was performed on a 1.5 Tesla MRI machine (Signa^TM^ Explorer General Electric, GE Healthcare, Fairfield, Waukesha, WI, USA; MR software release: SV25.2_2127a).

The scalp lesion had been managed for several years as eczema or infection with topical corticosteroids, antibiotics, and antifungal agents, but without significant improvement. Her serological and hematological tests—including complete blood count, inflammatory markers, and liver and renal function tests—showed no significant abnormalities, and no other specific serological tests were documented in her medical records.

**Figure 2:**
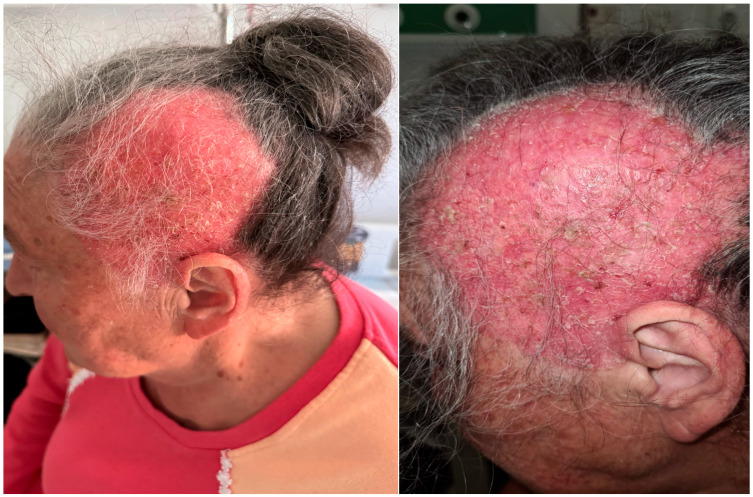
A 15 cm, red, shiny, atrophic plaque with scales on the left fronto-parieto-temporal region of a 70-year-old woman, representing the clinical presentation of extramammary Paget’s disease of the scalp.

In April 2024, she was referred to a private clinic where differential diagnoses of discoid lupus and intraepithelial neoplasia were considered. Antinuclear antibody (ANA) testing was performed and returned positive (titer: 1:640). A skin biopsy of the lesion revealed moderate epidermal dysplasia, but without the full histological features characteristic of Bowen’s disease. She was referred to our clinic in May 2024. On examination, a 15 cm red, shiny, atrophic area was noted on the left lateral side of her scalp, extending under normal white hair. The center of the lesion featured an alopecic zone with erosions, crusts, and yellow and white scales, though it was non-infiltrated (Figure 2). The rest of the clinical examination was unremarkable, with no palpable lymphadenopathy in the cervical, axillary, or inguinal regions. Additionally, the patient exhibited no signs of neurological involvement, such as nerve fiber abnormalities.

**Figure 3:**
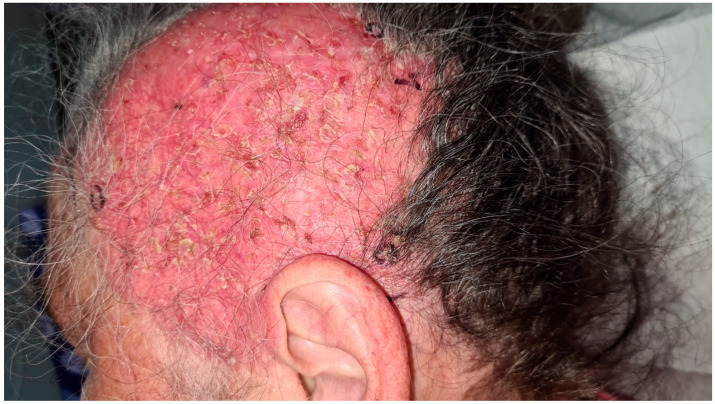

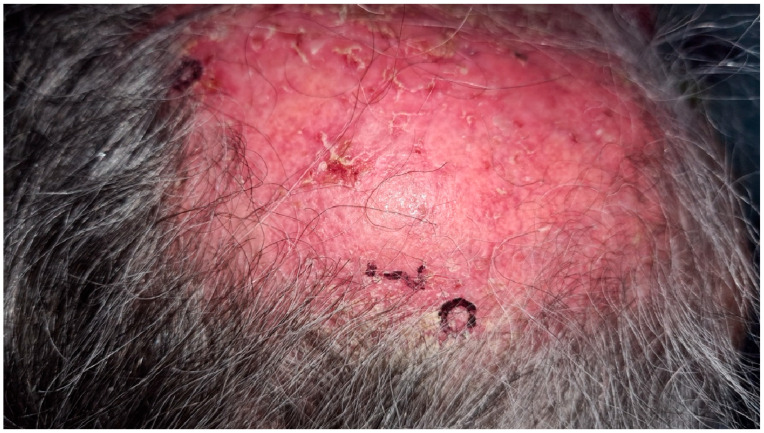
A 15 cm, red, shiny, atrophic plaque with scales on the left fronto-parieto- temporal region of a 70-year-old woman, representing the clinical presentation of extramammary Paget’s disease of the scalp and the biopsy area marked in black.

Laboratory evaluation demonstrated normal results for complete blood count, serum electrolytes, and assessments of kidney and liver function. Antinuclear antibody (ANA) testing was positive, with a titer of 1:2560. Nonetheless, the extractable nuclear antigen (ENA) antibody panel was negative in May 2024. Anti-desmoglein antibodies were also negative. Micological direct tests and cultures were negative. Four biopsies, including intact epidermis from the lesion’s edge, were taken (Figure 3).

**Figure 4:**
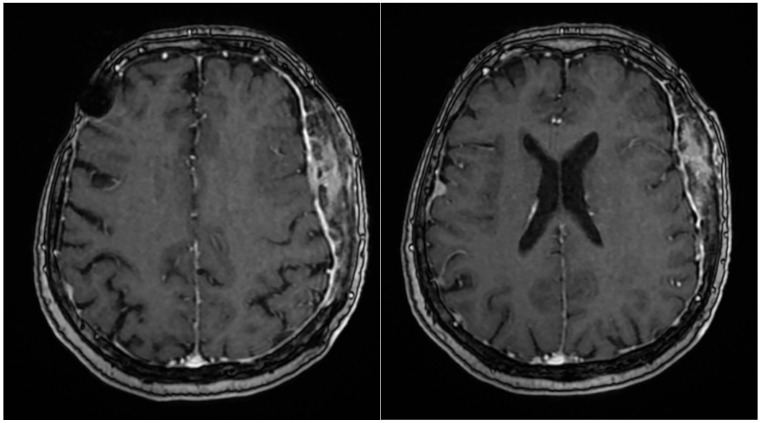
The same examination 16 months later revealed the same aspect, without significant changes.

Given her history of multiple meningiomas, a neurosurgery consultation was obtained. MRI revealed no progression of the previously identified meningiomas (Figure 4), and the skin lesion was found to be adjacent to the intraosseous meningioma. Skin ultrasound of the lesion demonstrated soft tissue infiltration consistent with meningiomatosis. Additional investigations, including mammography and a gynecological exam, showed no abnormalities.

**Figure 5:**
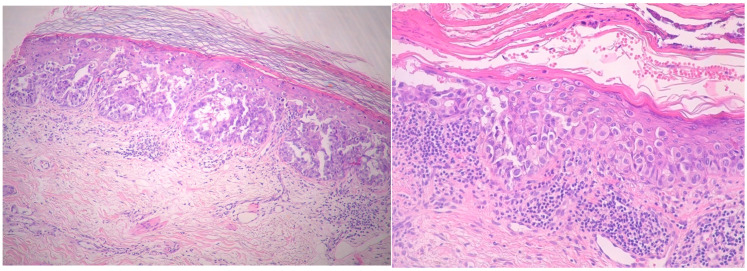
Histopathological picture. Large cells with clear cytoplasm and atypical nuclei, arranged on their own or in nests in the epidermis (Paget cells), spread partially to the adnexa, but with no dermal infiltration (hematoxylin and eosin, original magnification, ×10 ×20).

Histopathological examination of the biopsy specimens revealed skin covered by fibrino-leukocytic exudate with areas of epidermal ulceration. Medium-sized, atypical, discohesive tumor cells were observed in the epidermis and hair follicles, with pagetoid spread into the spinous layer. These atypical cells extended partially into the adnexal structures but did not infiltrate the dermis. The dermis showed moderate mixed perivascular inflammatory infiltrate and actinic elastosis (Figure 5).

**Figure 6:**
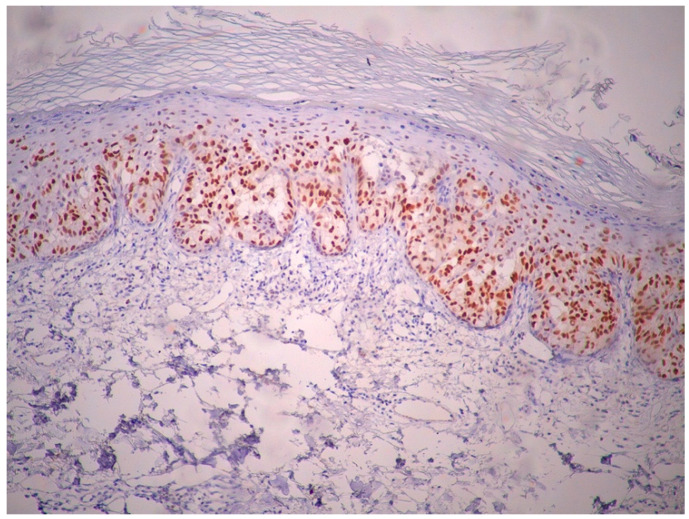
The nuclei of the atypical intraepidermal cells show strong and diffuse positive immunoreactivity for GATA3, supporting the diagnosis of Paget disease (magnification ×10).

**Figure 7:**
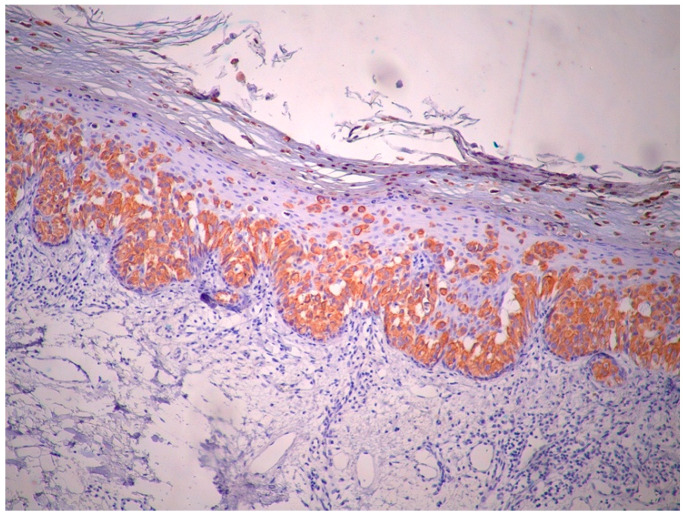
The immunohistochemical stain demonstrates strong, diffuse cytoplasmic positivity for CK7 in the atypical intraepidermal cells. This staining pattern confirms their epithelial nature and supports the diagnosis of EMPD (magnification ×10).

**Figure 8:**
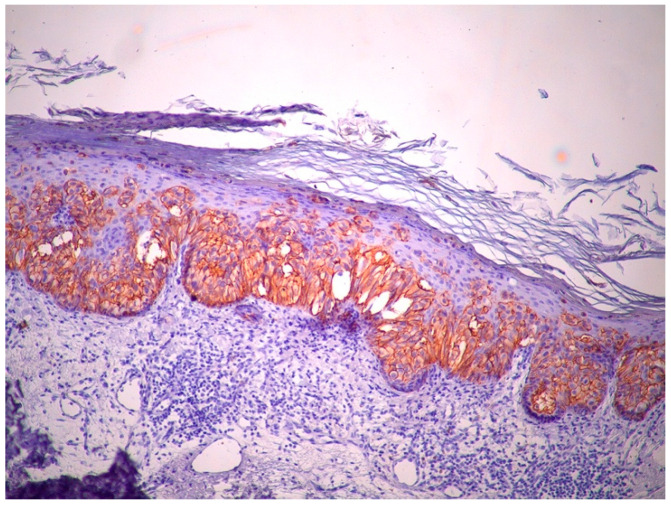
There is strong, circumferential membranous positivity for HER2 in the intraepidermal. Paget cells, indicative of HER2 overexpression (magnification ×10).

**Figure 9:**
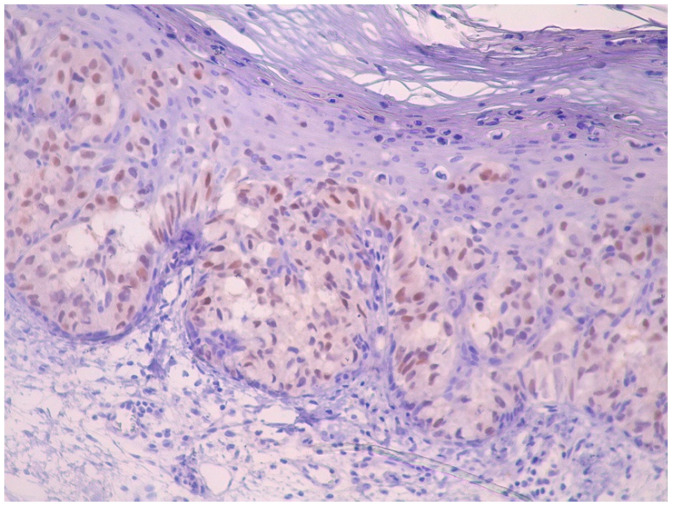
The Paget cells show weak and focal nuclear positivity for androgen receptor (AR), indicating limited expression (magnification ×10).

**Figure 10:**
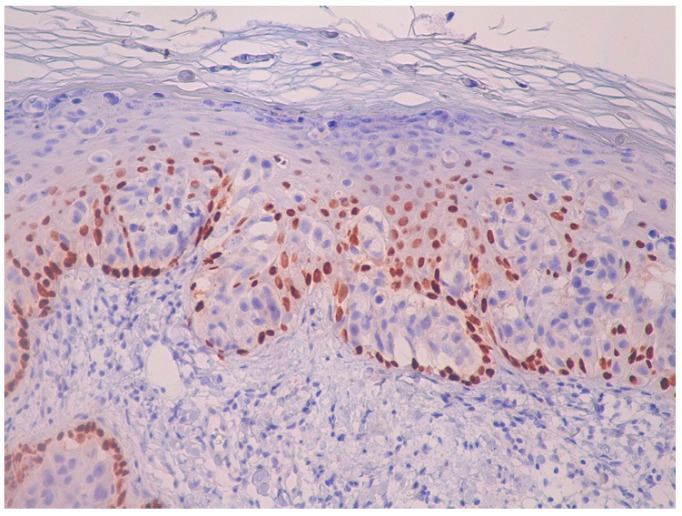
The atypical Paget cells show no nuclear staining for p63, confirming p63 negativity. In contrast, basal keratinocytes in the surrounding normal epidermis retain p63 positivity, serving as an internal positive control (magnification ×10).

**Figure 11:**
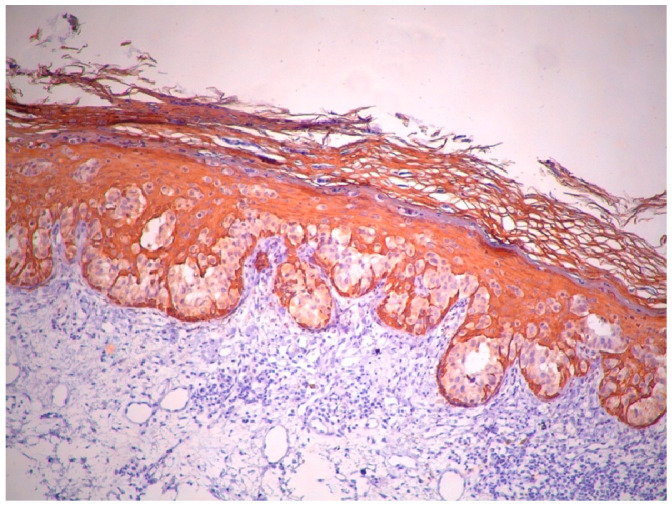
The atypical intraepidermal Paget cells demonstrate no cytoplasmic staining for CK5/6, indicating negative expression. Normal basal keratinocytes retain CK5/6 positivity, serving as an internal control. The absence of CK5/6 staining in the Paget cells helps differentiate EMPD from other intraepidermal neoplasms such as squamous cell carcinoma in situ (Bowen’s disease) and pagetoid variants of adnexal tumors, which typically express basal-type cytokeratins such as CK5/6 (magnification ×10).

Immunohistochemically, atypical cells were positive for GATA3 (Figure 6), CK7 (Figure 7), HER2 (Figure 8) and AR (Figure 9) and negative for p63 (Figure 10), CK5/6 (Figure 11) and CK20. Also, estrogen and progesterone receptors were negative.

**Figure 12:**
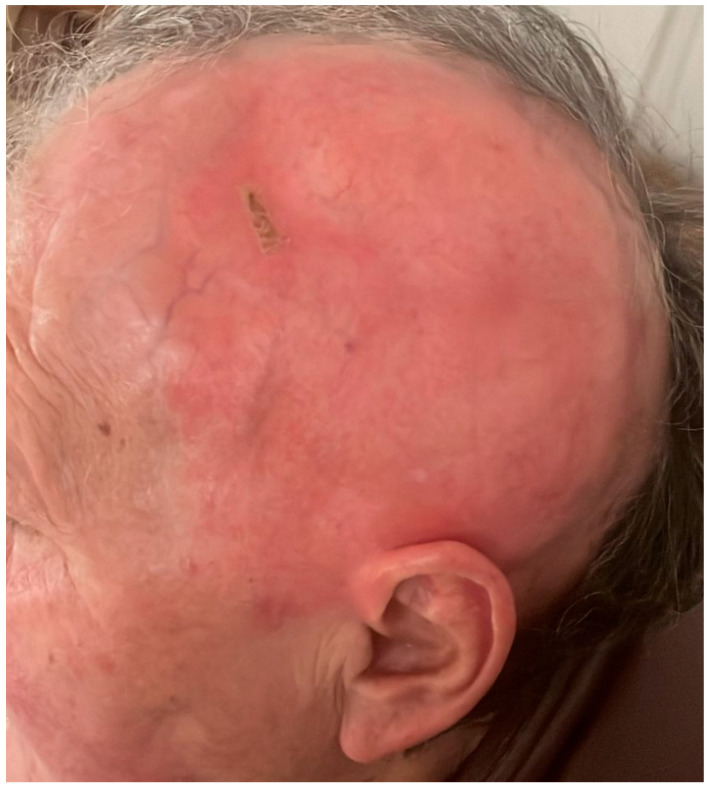
Clinical appearance of a 70-year-old female patient with extra-mammary Paget’s disease of the scalp following 25 sessions of radiotherapy. The image shows reduced erythema, resolution of scaling, and residual edema.

Based on these clinical and pathological findings, a diagnosis of ectopic EMPD of the scalp was made. No involvement of other organs was detected in either clinical or paraclinical evaluations. The intraosseous meningioma was surgically resected in the Department of Neurosurgery. Skull reconstruction was performed using an autologous periosteal graft and cranioplasty with a titanium mesh. Histopathological examination confirmed the diagnosis of a grade II atypical meningioma. Regarding the associated skin lesion, radiotherapy has been selected as the preferred treatment modality. The patient completed the 25 sessions of photon irradiation at the end of May 2025, with a favorable response to treatment. The lesion resolved, leaving only residual edema, as shown in Figure 12.

## 3. Discussion

Extramammary Paget’s Disease (EMPD) is a heterogeneous condition that may represent either an in situ apocrine carcinoma or a manifestation of an underlying primary skin adenocarcinoma or internal organ malignancy, particularly in the anogenital region [7].

Typically, EMPD presents as pruritic, erythematous, eczematous plaques in the anogenital area. However, variations such as depigmented plaques, papillomatosis, lichenification, ulceration, or bleeding have also been reported [8,9]. Ectopic extramammary Paget disease (EMPD) is a rare, slow-growing neoplasm that arises in areas not typically bearing apocrine glands. We conducted a systematic search in PubMed using the keywords “ectopic” and “extramammary Paget disease.” Additionally, we reviewed the reference lists of the included articles to identify further relevant studies. Around 30 cases of EMPD occurring outside the perianal, genital, and axillary regions have been documented in the English-language literature [10]. To the best of our knowledge, only nine cases of ectopic EMPD involving the scalp have been published in the English-language literature (Table 1).

EMPD occurs in both males and females, with a modest predominance in females, as reflected in the table showing a female-to-male ratio of 5:4. A recent study reported the average age at diagnosis to be 74 years for invasive EMPD and 72 years for non-invasive cases. The mean age of the EMPD of the scalp cases is 62.7 years [12,17,18].

With respect to tumor location, two out of nine cases (22.2%) were identified in the right parietal region, one case (11.1%) in the right frontotemporal area, and two cases (22.2%) in the occipital region. The exact location was not specified for the remaining cases. Tumor sizes ranged from 1 cm to 8.5 × 6 cm in diameter, with our case presenting the largest size, measuring 15 cm in diameter. Associated hair loss/alopecia was reported in four other cases [12,14,15,16].

In our case, extramammary Paget’s disease was considered as a differential diagnosis due to its clinical resemblance to eczema, presenting as a slowly enlarging, reddish area with crusts, mimicking an eczematous lesion. Additional differential diagnoses considered included superficial pemphigus, chronic lupus erythematosus, pustular erosive dermatoses, Bowen’s disease, cicatricial pemphigoid, basal cell carcinoma, cutaneous T-cell lymphoma, and neurotrophic lesions. In the frontotemporal area, EMPD can also clinically resemble seborrheic dermatitis, nummular eczema, and, less commonly, lichen sclerosus et atrophicus and lichen simplex chronicus [6,12].

Histopathological examination is a crucial component in diagnosing ectopic EMPD, as it helps differentiate EMPD from other epidermal malignancies. Paget cells, which are large, round cells with abundant pale basophilic cytoplasm and large centrally located nuclei, are present in the epidermis and can rarely invade the dermis [12].

Immunohistochemical markers such as CK7 usually show high expression in EMPD lesions. One study of 15 EMPD cases found all cases positive for CK7 and negative for CK20 [19]. In contrast, a study by Liegl et al., involving 23 EMPD cases, reported CK7 positivity in 100% (23/23) and CK20 positivity in 13% (3/23) [20]. Another study of 28 EMPD cases by Diaz et al. identified androgen receptor (AR) positivity in 15 of 28 cases, while all cases lacked estrogen receptor (ER) and progesterone receptor expression [21]. The p63 gene and CK5/6 markers are useful in distinguishing EMPD from the pagetoid variant of Bowen’s disease. In our case, similar to other reports, the tumor was positive for CK7 and AR, and negative for CK20 and p63 [14].

In our case, laboratory findings revealed a positive ANA titer (1:2560), while the panel of extractable nuclear antigen antibodies (ENAs) was negative. To date, no association between elevated ANA titers and EMPD has been reported in the literature. However, the presence of ANA is nonspecific and can be linked to various non-autoimmune factors, such as carcinomas, infections, medications, and environmental influences. Studies suggest that the frequency of ANA positivity in healthy individuals is approximately 20% [22,23].

While mammary Paget’s disease is typically associated with an underlying breast carcinoma, when diagnosing EMPD, the patients should be screened for underlying malignancy [12,24]. EMPD is reported to be associated with other malignancies in the range 16–48% [24,25,26,27]. In a retrospective study of 197 patients, Chanda found an incidence of 24% for underlying adnexal carcinoma and 12% for concurrent internal malignancies [17]. More recently, Lai et al. reported a 21% incidence of associated adnexal carcinoma and 9% for underlying internal malignancies [28].

The connection between EMPD and invasive cancer is intricate and might indicate differences in tissue origin. In the majority of instances, Paget cells remain restricted to the epidermis and the epithelium of adnexal structures [16]. Nevertheless, as many as 25% of cases involve malignancies that invade the dermis, frequently originating from apocrine or eccrine glands. Interestingly, there has been a documented case of EMPD on the scalp associated with an apocrine hidradenocarcinoma [7].

In previously reported cases of ectopic EMPD on the scalp, most of the cases were not associated with any subsequent lesions (66.6%) [5,6,7,11,12,13,14,15,16]. In three cases, associations have been identified with various tumors: one case involved hidradenocarcinoma showing mucinous differentiation and renal cell carcinoma (11.1%) [7], another was associated with nodular basal cell carcinoma (11.1%) [16] and the third case was associated with a seborrheic keratosis (11.1%) [5]. Interestingly, in our case, we did not observe any association with a malignancy; however, it was linked to an intraosseous atypical meningioma.

Different subtypes of EMPD have varying prognostic outcomes. Lesions without dermal invasion or associated visceral malignancies have a high rate of local recurrence but relatively good overall prognosis [16]. Conversely, ectopic EMPD associated with underlying visceral malignancies carries a much worse prognosis, with a mortality rate exceeding 50% [16,29,30].

In general, EMPD is only locally aggressive. A variety of treatment options exist; however, surgical resection is the treatment of choice [14]. However, recurrence rates are high even when the lesions are removed with wide surgical margins [14]. Lower recurrence rates have been reported with to Mohs micrographic surgery in comparison with standard excision [31].

In previously reported cases of EMPD of the scalp (Table 1), the treatment of choice in the majority of cases (66.6%) was surgical excision [5,7,11,13,14,16]. Only one case (11.1%) involved the use of Mohs surgery [12], while two cases (22.2%) were treated with photodynamic therapy [6,15].

Most recent studies have suggested safety margins of 2 to 5 cm when performing wide local excision [31,32,33]. However, in a separate study, 5 out of 66 patients who had curative surgery experienced local recurrence, and no significant link was found between the size of the surgical margin (whether ≤2 cm or >2 cm) and recurrence rates [34]. These findings indicate that a surgical margin of 1 to 2 cm may be sufficient for wide local excision in the treatment of EMPD. In the case reported by Kuniyuki et al. and Debarbieux et al., a wide local excision with 2–3 cm and 1.5 cm margins, respectively, was performed [13,14]. However, recurrence rates remain high even when wide surgical margins are used. In the case reported by Son et al. a wide local excision with 2 cm safety margins of the lesion was performed, but the recurrence appeared 2 years after [11]. Mohs micrographic surgery has been associated with lower recurrence rates compared to standard excision. Recurrence is likely due to the multifocal nature of the disease and subclinical involvement of apparently unaffected skin [31,35,36]. Although Mohs surgery improves cure rates, it does not completely prevent recurrence, especially in cases of invasive disease [6]. As regarding the present case, the treatment of choice was the surgically resection of the intraosseous meningioma in the Department of Neurosurgery and skull reconstruction afterwards. Regarding the associated skin lesion the preferred treatment approach was radiotherapy.

In cases where surgery cannot be applied, radiotherapy can be effective for EMPD primary lesions. Hata et al. demonstrated that the 5-year local progression-free rate after radiation therapy was 82% [37]. Moreover, radiation therapy can be useful as adjuvant treatment for patients at high risk of recurrence. In another study, Hata et al. found that no patients showed local recurrence after adjuvant radiotherapy, at a median follow up period of 38 month [38]. Radiotherapy has shown excellent results for intraepithelial (in situ) EMPD; however, in cases of invasive EMPD treated with radiation, recurrence rates can be as high as 50% [39]. Other non-surgical treatment options include imiquimod, topical 5-fluorouracil, topical bleomycin, systemic chemotherapy, laser therapy, and photodynamic therapy (PDT) [40,41,42].

PDT is particularly useful when surgery is not feasible, as EMPD often extends beyond the clinically visible tumor margins. In a case report by Cordoba et al., photodynamic therapy was administered in two sessions, one week apart, using topical methyl aminolevulinate. At the two-month follow-up, complete remission was observed, but partial recurrence occurred 18 months later. A second cycle of two PDT sessions resulted in partial remission, and imiquimod was added to the treatment regimen at the patient’s last visit [6]. Another case report by Al Youssef et al. described PDT using methyl 5-aminolevulinate in three sessions administered at four-week intervals. Almost complete remission was achieved after three months, with a full clinical response maintained 12 months after the final PDT session [15]. Due to the limited number of studies and case reports involving PDT in EMPD, this therapy should be considered only when other treatment options are not viable. In cases of advanced or metastatic EMPD, systemic chemotherapy is indicated. Unfortunately, patients with distant metastases tend to have a poor outlook, as standard chemotherapy treatments commonly employed for EMPD demonstrate limited effectiveness [43].

Continuous follow-up is essential to enable early detection of recurrence, which may present with a delayed onset. For non-invasive EMPD, follow-up visits are recommended twice a year for the first three years, followed by annual monitoring for up to 10 years [1]. In cases of invasive EMPD, or when associated with distant tumors, more frequent follow-up is advised—three to four times per year—with biopsies performed for any suspicious skin lesions [1].

## 4. Conclusions

We present an unusual case of ectopic EMPD of the scalp associated with an intraosseous meningioma. This represents an extremely rare location for ectopic EMPD, marking only the 10th documented case in this region, and the largest reported lesion to date. Notably, the association between ectopic EMPD and intraosseous meningioma has not been previously described. We emphasize that in cases of nonspecific lesions on the scalp, ectopic EMPD should be considered in the differential diagnosis. Ectopic EMPD is a rare condition, and the risk of associated malignancies remains poorly characterized, warranting thorough screening for underlying malignancies in EMPD patients. Further research is necessary to better understand the potential associations between ectopic EMPD and other malignancies, as well as to improve management and treatment strategies.

## Figures and Tables

**Table 1:** Case reports of ectopic EMPD of the scalp.

Author	Age/Sex	Location. Size	Associaeted Lesion	Treatment
Son et al., 2002 [11]	45/Female	NR/5 cm	-	Surgical excision
Hans Iwenofu et al., 2008 [12]	57/Female	Right parietal/5 × 3 cm	-	Mohs micrographic surgery
Wahl et al., 2009 [7]	54/Male	NR/1 cm	Hidradenocarcinoma with mucinous differentiation Renall cell carcinoma	Surgical excision
Cordoba et al., 2013 [6]	64/Female	Right frontotemporal/5 cm		Photodynamic therapy + imiquimod
Debarbieux et al., 2014 [13]	54/Male	Posterior vertex/6 cm	-	Surgical excision
Kuniyuki et al., 2015 [14]	82/Male	Occipital/85 × 50 mm	-	Surgical excision
Yourssef et al., 2015 [15]	86/Male	Right parietal/8.5 × 6 cm	-	Photodynamic therapy
Kang A. et al., 2016 [16]	73/Female	NR/20 mm	Nodular Basal Cell Carcinoma	Surgical excision
Balamoti et al., 2017 [5]	50/Female	NR/2 × 2 cm	Seborrheic keratosis	Surgical excision

## Data Availability

No new data were created or analyzed in this study. Data sharing is not applicable to this article.
